# Novel Biological Hydrogel: Swelling Behaviors Study in Salt Solutions with Different Ionic Valence Number

**DOI:** 10.3390/polym10020112

**Published:** 2018-01-24

**Authors:** Yu Wang, Guidong He, Zheng Li, Jiachuan Hua, Maoqi Wu, Jixian Gong, Jianfei Zhang, Li-tong Ban, Liang Huang

**Affiliations:** 1 College of Agronomy and Resources Environment, Tianjin Agricultural University, Tianjin 300384, China; banlitong@126.com (L.t.B.); huangliang@tjau.edu.cn (L.H.); 2 Key Laboratory of Advanced Textile Composites (Tianjin Polytechnic University), Ministry of Education; School of Textiles, Tianjin Polytechnic University, Tianjin 300387, China; calvinheguidong@163.com (G.D.); huajiachuan@sina.com (J.H.); wwumaoqi@163.com (M.W.); gongjixian@163.com (J.G.); zhangjianfei1960@126.com (J.Z.); 3 Key Laboratory of Science & Technology of Eco-Textile, Donghua University, Shanghai 201620, China

**Keywords:** poly γ-glutamic acid, ε-polylysine, hydrogels, swelling behaviors

## Abstract

In this paper, poly γ-glutamic acid/ε-polylysine (γ-PGA/ε-PL) hydrogels were successful prepared. The γ-PGA/ε-PL hydrogels could be used to remove Na^+^, Ca^2+^, and Cr^3+^ from aqueous solution and were characterized by scanning electron microscopy. The performance of hydrogels were estimated under different ionic concentration, temperature, and pH. The results showed that the ionic concentration and the pH significantly influenced the swelling capacity of γ-PGA/ε-PL hydrogels. The swelling capacities of γ-PGA/ε-PL hydrogels were decreased with the increase of the ionic concentration. However, the swelling capacity of the γ-PGA/ε-PL hydrogel was increased with the increase of the pH. The swelling kinetics indicated that γ-PGA/ε-PL hydrogels presented a more limited swelling degree in metal ion solutions with higher ionic valence numbers than in ion solutions with lower ionic valence numbers. However, the swelling kinetics of γ-PGA/ε-PL hydrogels showed that they proposed a satisfactory description in NaCl and CaCl_2_ solutions. The adsorption process was fitted with a pseudo-second-order rate equation model. Moreover, the desorption kinetics of γ-PGA/ε-PL hydrogels showed that they could release most of the adsorption ions. Considering the biocompatibility, biodegradability, and ionic-sensitive properties, we propose that these γ-PGA/ε-PL hydrogels have high potential to be used in environmental protection, medical treatment, and other related fields.

## 1. Introduction

During the past decades, hydrogels, as a kind novel functional material, have been the material of choice for many applications due to their unique biocompatibility, flexible methods of synthesis, desirable physical characteristics, adjustable biomimetic properties, and absorbing ability [1,2]. They can serve in tissue engineering [3,4], wound-dressing [5,6], drug delivery systems [7,8], superabsorbents [9,10] and many other related branches of study. Natural polymers and synthetic polymers could be used to prepare hydrogels [11]. However, more and more novel hydrogels with biodegradability and stimulus responsive have been prepared from natural polymers in recent years [12,13,14].

Our society has suffered a great deal from industrial ion wastewater pollution. Ion wastewater is one of the most serious environment problem, globally. Even in very low concentration, metal ions, such as Cu^2+^, Cr^3+^, and Cd^2+^, can accumulate in the environment and/or living tissues, generating various diseases and abnormalities in living organisms [15,16,17,18]. Thus, removal of metal ions from the aquatic environment is a vital issue. Many researchers have focused on the production of effective methods for removing metal ions from wastewater, including chemical precipitation, ion-exchange, electrochemical treatment, membrane separation, and others [19]. However, these techniques have some disadvantages, such as inefficiency, high-cost, the formation of toxic sludge, and other waste products [20]. Hence, developing cheaper, efficient, and environmentally-friendly adsorbents and technologies are attracting the attention of researchers [21]. Hydrogels, as a kind of unique adsorbent, have the potential for effective ion removal and have receive increasing attention compared with conventional counterparts [22].

Hydrogels, which are defined as a three-dimensional polymer network composed of cross-linked hydrophilic polymer chains and hold a significant amount of aqueous solvent [11]. As a new type of adsorbent, hydrogels can be practically made into any shape or size [23,24]. Hydrogel can absorb liquids to reach 1000-fold of their dry weight because of the hydrophilic groups of the network and retain important fractions of water within its structure, but they do not dissolve [25]. Among natural polymers, poly γ-glutamic acid (γ-PGA) has gained significant attention to the preparation of hydrogels. γ-PGA is a non-poisonous, esculent, and anionic polypeptide biomaterial [26]. Now, it can be mass produced by microbial fermentation [27]. Similarly, natural ε-polylysine (ε-PL) is also biodegradable, esculent, and non-poisonous towards humans, and is environmentally-friendly [14,28]. ε-PL is a cationic polyamide biomaterial that is composed of ε-NH_2_-α-COOH with an l-lysine linkage and has antibacterial properties [29,30]. 

In this study, we prepared a type of novel γ-PGA/ε-PL hydrogel by EDC/NHS mediated polymerization. We studied the effects of pH, temperature, ion concentration, and ion valence number on the swelling characteristics of γ-PGA/ε-PL hydrogels. The pseudo-first-order and pseudo-second-order equations were utilized to verify the mechanism and kinetics of the swelling process. At the same time, the desorption kinetics of γ-PGA/ε-PL hydrogels was also investigated.

## 2. Materials and Methods

### 2.1. Materials

Poly γ-glutamic acid was provided from Shineking Biotechnology (Nanjing, China). ε-Polylysine was offered from Silver-Elephant Bio-engineering Co. (Zhejiang, China). 4-Morpholineethanesulfonic acid (MES), *N*-hydroxysuccinimide (NHS), and 1-ethyl-3-(3-dimethylaminopropyl) carbodiimide hydrochloride (EDC·HCl) were supplied from Sinopharm Chemical Reagent Co. (Shanghai, China), respectively. NaCl, CaCl_2_, and CrCl_3_·6H_2_O were purchased from Sinopharm group Co., Ltd. (Shanghai, China).

### 2.2. Preparation of γ-PGA/ε-PL Hydrogels

γ-PGA/ε-PL hydrogels were prepared by the EDC/NHS mediated system and the molar ratio of γ-PGA:ε-PL:EDC:NHS was 1:0.1:0.25:0.25 [14]. Firstly, 0.1 mol/L MES solution were prepared by deionized water. Then, γ-PGA and ε-PL were dissolved into pre-made 0.1 mol/L MES solution, respectively. After that, the γ-PGA/ε-PL solution was obtained by slowly adding ε-PL solution into γ-PGA solution and stirring at 100 r/min for 15 min, shifting it into a 0 °C refrigerator for 40 min. Afterward, the NHS was dissolved into the γ-PGA/ε-PL solution and EDC was added in order. Finally, the γ-PGA/ε-PL hydrogel could be quickly made after stirring at 100 r/min for 10 min [14]. 

### 2.3. Swelling Study

**Figure 1:**
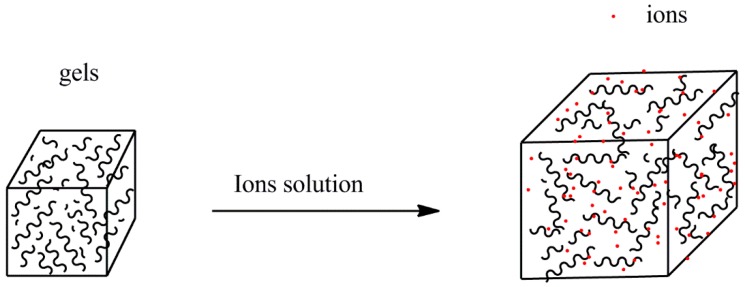
The simulation of the swelling of γ-PGA/ε-PL hydrogels in ion solutions.

The swelling study was conducted in three different concentrations of Na^+^, Ca^2+^, and Cr^3+^ solutions (as shown in Figure 1). γ-PGA/ε-PL hydrogel cubes were dried in a drying oven at 50 °C. Then, dried hydrogels were dipped into 200 mL Na^+^, Ca^2+^, and Cr^3+^ solution, respectively, to equilibrium at room temperature. The weights of the wet hydrogels (*W*_0_) were evaluated at 24 h. The wet hydrogels were then completely dried at 50 °C and the weights of dry hydrogels (*W*_d_) were evaluated [31,32]. The swelling degree (*Q*) is expressed by Equation (1):

(1) $$Q=\left(W_{0}-W_{d}\right)/W_{d}$$

#### 2.3.1. The Effects of pH and Temperature

The effect of pH on swelling characteristics was studied by adding the dried hydrogels into 200 mL 0.0154 mol/L ion solutions. The ions solutions were adjusted on the pH values (pH 1–7.0 for Na^+^ and Ca^2+^, 1–5.0 for Cr^3+^) by hydrochloric acid and/or sodium hydroxide. The solutions were placed at room temperature for 24 h. To study the effect of temperature on swelling characteristics, the dried hydrogels were added into 200 mL 0.0154 mol/L ion solutions and in an incubator at 15, 30, 45, and 60 °C for 24 h.

#### 2.3.2. The Swelling Kinetic Study

The swelling kinetic was tested at different concentrations of Na^+^, Ca^2+^, and Cr^3+^ solutions. γ-PGA/ε-PL hydrogels were dried at 50 °C. The dried hydrogels were dipped into 200 mL Na^+^, Ca^2+^, and Cr^3+^ solutions to equilibrium at room temperature. The swelling rate in salt solution of γ-PGA/ε-PL hydrogels could be calculated by pseudo-first-order and pseudo-second-order kinetics models. The two models were expressed from the following Equations (2) and (3), respectively [33,34]:

(2) $$\mathrm{lg}\left(Q_{e}-Q_{t}\right)=\mathrm{lg}Q_{e}-\frac{K_{1}t}{2.303}$$

(3) $$\frac{t}{Q_{t}}=\frac{1}{K_{2}Q_{e}^{2}}+\frac{t}{Q_{e}}$$

where *Q*_t_ (g/g) and *Q*_e_ (g/g) are the amount of swelling in equilibrium and at time *t*; *K*_1_ (min^−1^) and *K*_2_ (min^−1^) are the rate constant of the pseudo-first-order and pseudo-second-order kinetics models, respectively.

### 2.4. The Desorption Kinetic

**Figure 2:**
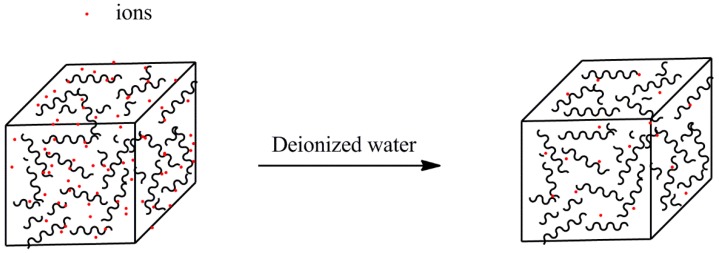
The simulation of desorption of γ-PGA/ε-PL hydrogels in deionized water.

The desorption kinetics were carried out in deionized water at room temperature (as shown in Figure 2). In order to prepare ion loaded hydrogels, the hydrogels were immersed in 100 mL 0.0154 mol/L ion solutions for 24 h until equilibrium. Then, the ion-loaded gels were taken out and dried in a drying oven until constant weight. Dry gels were soaked in deionized water (50 mL) at room temperature. The conductivity was recorded by a conductivity meter. Then, the conductivity was transformed into the ion concentration by using the standard curve obtained with a series of standard ion solutions [35].

## 3. Results and Discussion

### 3.1. Preparation of Hydrogels

γ-PGA/ε-PL hydrogels were prepared by the EDC/NHS mediated system. As shown in Table 1, after swelling equilibrium, γ-PGA/ε-PL hydrogels displayed a similar color as those of the ion solutions. This indicated that swelled hydrogels adsorbed metal ions into their structure. Then, the cross-section and surface morphology of γ-PGA/ε-PL hydrogels were visualized by SEM (TM-3030, Hitachi, Japan). γ-PGA/ε-PL hydrogels showed a three dimensional network and the porous structure of hydrogels was covered with irregularly-shaped holes [14]. With the ionic valence number increased, the network structures became smaller and presented a slight collapse. While swelled in CrCl_3_ solution, the porous structure in the hydrogels almost completely collapsed. These phenomena indicated that increasing the ionic valence number could hinder the swelling behavior of γ-PGA/ε-PL hydrogels.

### 3.2. Swelling Study

#### 3.2.1. The Effects of pH and Temperature

**Figure 3:**
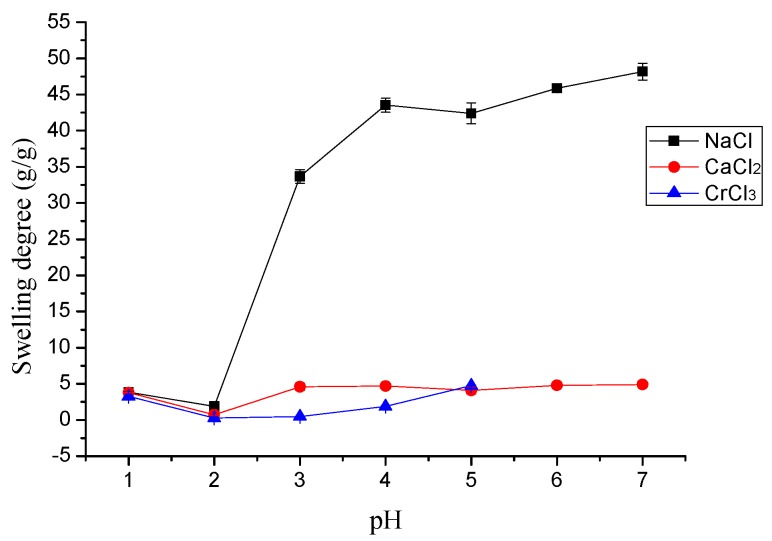
The effect of pH on the swelling study of metal ions.

The effect of pH on the swelling behavior of γ-PGA/ε-PL hydrogels are shown in Figure 3. In general, high pH increased the swelling capacity of the hydrogel in ion solutions. However, the swelling degree was low at lower pH and the swelling degree was at a minimum at pH 2.0. The swelling degree in NaCl and CaCl_2_ solutions were suddenly decreased at pH 5.0. This phenomenon was attributed to the principle that the –NH^3+^ and –COO^−^ groups within the network of γ-PGA/ε-PL hydrogels were protonated at a lower pH and led to a pH-sensitivity. The metal ions had to compete with the protons for absorbency and protonated functional groups hindered the interaction between the γ-PGA/ε-PL hydrogels and metal cations [36]. Obvious metal hydroxide precipitate occurred when the pH increased to 6.0 for Cr^3+^. Hence, the optimal pH for Cr^3+^ adsorption was 5.0.

**Figure 4:**
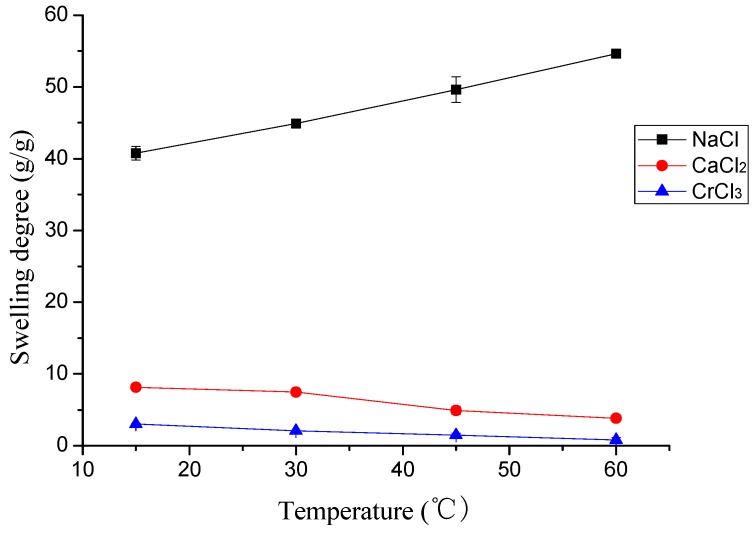
The effect of temperature on the swelling study of metal ions.

Figure 4 describes the effect of temperature (pH 6.0) on the swelling behavior of γ-PGA/ε-PL hydrogels. In general, the swelling degree of γ-PGA/ε-PL hydrogels in NaCl solution was slightly raised with the temperature increasing from 15 to 60 °C. However, in CaCl_2_ and CrCl_3_ solutions, the swelling degree of γ-PGA/ε-PL hydrogels were slightly lower with the increase of temperature. This phenomenon was attributed to the effect of temperature and ion shielding effect. The influence of the ion shielding effect on the swelling degree was greater than that of the temperature in CaCl_2_ and CrCl_3_ solutions. In fact, the results showed that swelling behavior of γ-PGA/ε-PL hydrogels in ion solutions was barely influenced by the temperature. Compared to poly(*N*-hydroxyethylacrylamide) hydrogels, γ-PGA/ε-PL hydrogels presented a similar phenomenon in NaCl solution, but the opposite phenomenon in CaCl_2_ and CrCl_3_ solutions [36].

#### 3.2.2. The Swelling Kinetic Study

##### 3.2.2.1. The Effect of Ion Valence Number

**Figure 5:**
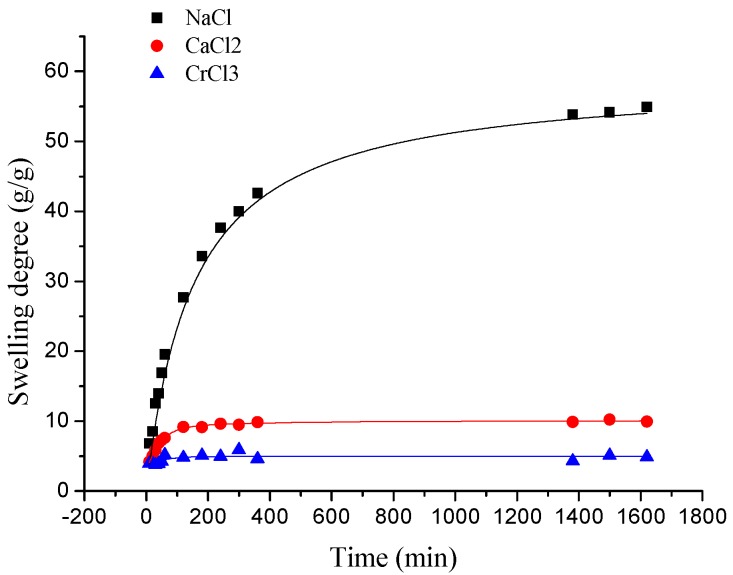
The swelling kinetics of γ-PGA/ε-PL hydrogels in NaCl, CaCl_2_, and CrCl_3_ solutions.

As presented in Figure 5, the swelling kinetic of γ-PGA/ε-PL hydrogels in NaCl, CaCl_2_, and CrCl_3_ solutions were different. At the beginning, the swelling degree increased rapidly. At approximately 120 min, the elevation of swelling degree was slow in both NaCl and CaCl_2_ solutions, and it decreased slightly in CrCl_3_ solution. In the final stage, the swelling degrees remained steady in various solutions. Finally, the swelling degrees of γ-PGA/ε-PL hydrogels in NaCl, CaCl_2_, and CrCl_3_ solutions were 54.91, 9.94, and 4.86 g/g, respectively.

**Figure 6:**
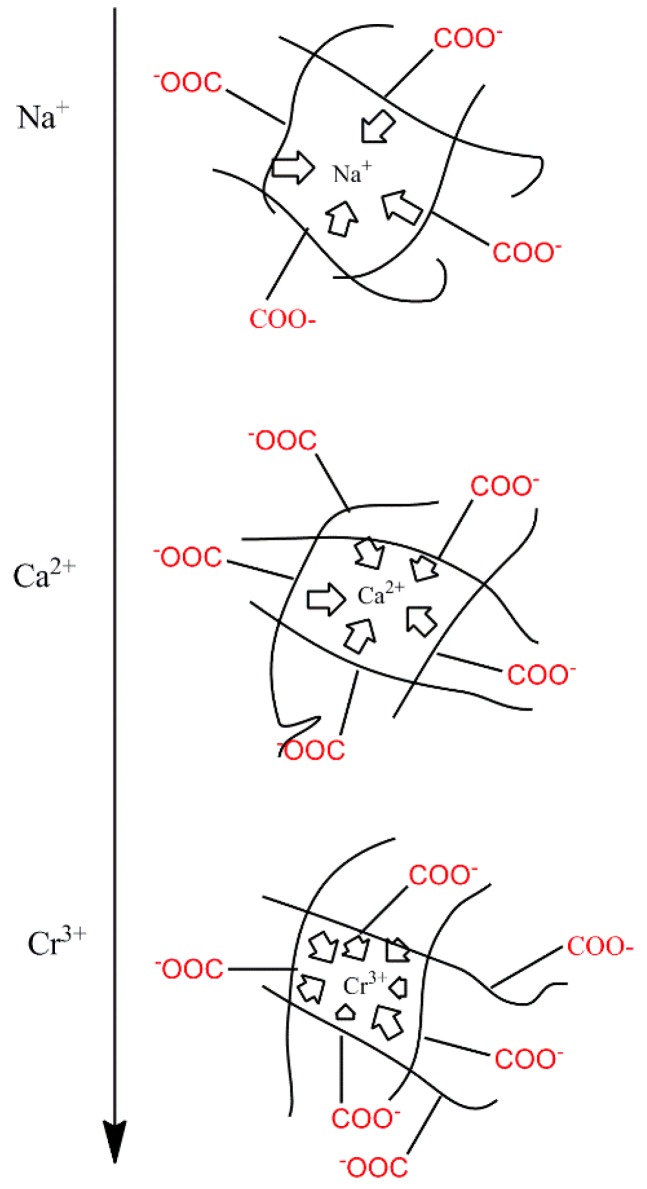
A schematic diagram of ionic charge shielding effect.

Compared to the swelling in ion solution of poly (aspartic acid) hydrogels, the swelling of γ-PGA/ε-PL hydrogels also showed the same trend [37]. This phenomenon was due to the chemical structure of γ-PGA/ε-PL hydrogels that contained a vast amount of –COOH and –NH_2_ in the polymeric network [14]. Those dissociative groups produced mutual adsorption with ions, leading to mutually exclusivity of the –COO^−^, and the swelling degree was restricted. As a result, γ-PGA/ε-PL hydrogels were more ion-sensitive and showed more limited swelling kinetics in ion solutions with higher ionic valence numbers, as shown in Figure 6.

##### 3.2.2.2. The Effect of NaCl Concentration

In order to investigate the effect of NaCl concentration on the swelling kinetic of γ-PGA/ε-PL hydrogels, the swelling degrees of the hydrogels were measured within the scheduled time and the swelling kinetics were fitted with adsorption kinetic models. Among numerous established adsorption kinetic models, the pseudo-first-order and pseudo-second-order kinetic models were the widely used and appropriated for study the swelling kinetic in ion solutions, such as Na(I), Ca(II), Cu(II), and Cr(IV) ion solutions [34,38,39].

**Figure 7:**
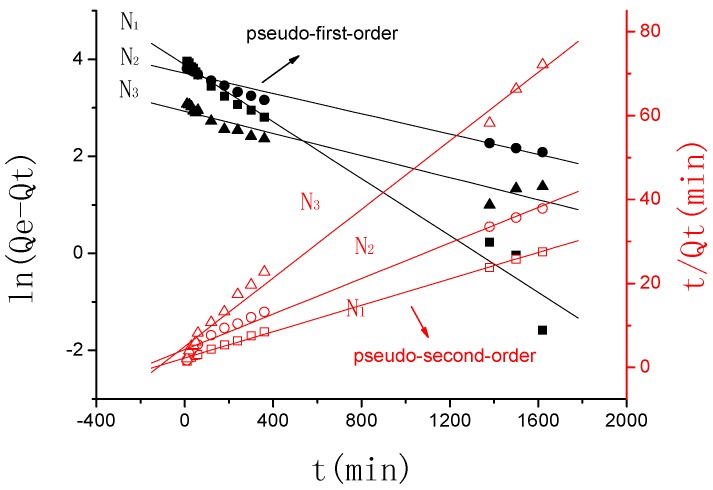
Pseudo-first-order and pseudo-second-order kinetic plot for the adsorption of γ-PGA/ε-PL hydrogel in NaCl solution.

Figure 7 describes the plots for the pseudo-first-order and pseudo-second-order kinetics of γ-PGA/ε-PL hydrogels in N_1_, N_2_, and N_3_ (N_1_: 0.0154 mol/L, N_2_: 0.0308 mol/L, N_3_: 0.154 mol/L). Table 2 lists the kinetic parameters of the pseudo-first-order and pseudo-second-order models. From Table 2, it can be seen that the linearly dependent coefficient (*R*_adj_^2^) for the pseudo-first-order model was very high. However, there were great differences between the experimental swelling degree (*Q*_e,e_) and the calculated swelling degree (*Q*_e,c_), which indicates that the pseudo-first-order model was unfit for the adsorption processes of γ-PGA/ε-PL hydrogels. However, the pseudo-second-order model revealed relatively higher linearly-dependent coefficients, which were all over 0.99. Moreover, the value of the experimental swelling degree (*Q*_e,e_) w closer to the calculated swelling degree (*Q*_e,c_). These showed that the adsorption processes of γ-PGA/ε-PL hydrogels and the composites for Na(I) could be perfectly expressed by the pseudo–second order model [34,36].

**Figure 8:**
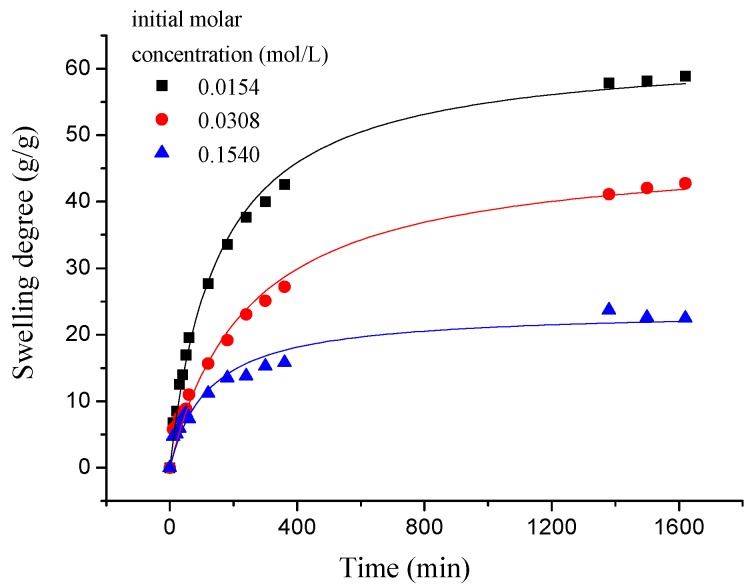
The swelling kinetics of γ-PGA/ε-PL hydrogels in NaCl solution.

As shown in Figure 8, the swelling kinetics of γ-PGA/ε-PL hydrogels in both concentrations expressed similar trends. Under the condition of the same concentration, adsorption capacities of ions increased rapidly and steadily with the increase of time (less than 800 min). At higher contact times, the increase in uptake slowed. However, the adsorption capacities of ions was decreased obviously with the increased of NaCl concentration, which indicated that the adsorption process was high concentration dependence manner [21,40]. This phenomenon is consistent with previous studies [14,41]. Compared to γ-PGA or ε-PL hydrogels, γ-PGA/ε-PL hydrogels presented good swelling capacities and a higher swelling degree in NaCl solution. This was due to the fact that γ-PGA/ε-PL hydrogels contained a mass of bonds of –CONH–, which could obviously mitigate the charge shielding effect of ions in the hydrogel’s network [14]. 

##### 3.2.2.3. The Effect of CaCl_2_ Concentration

**Figure 9:**
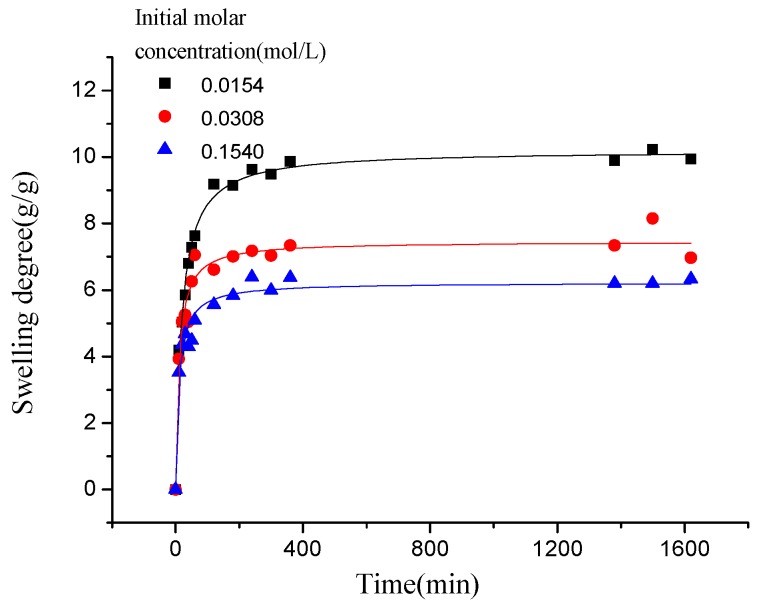
The swelling kinetics of γ-PGA/ε-PL hydrogels in CaCl_2_ solution.

**Figure 10:**
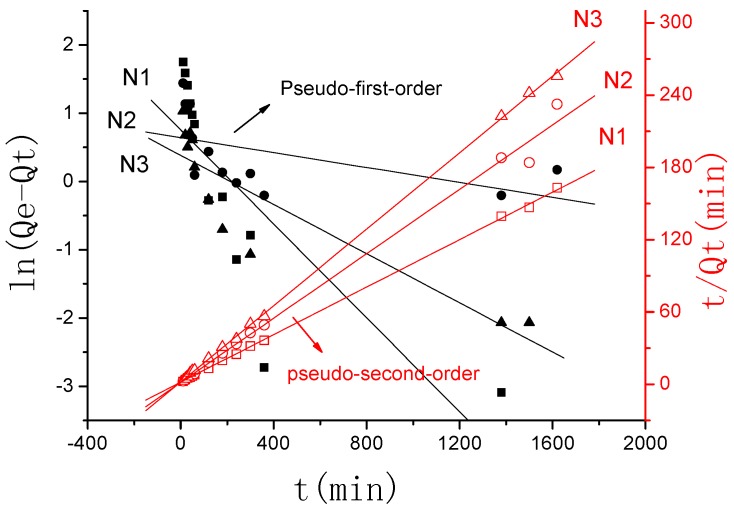
Pseudo-first-order and pseudo-second-order kinetic plot for the adsorption of γ-PGA/ε-PL hydrogels in CaCl_2_ solution.

Similar to the adsorption kinetic in NaCl solution, as shown in Figure 9, the adsorption kinetics of γ-PGA/ε-PL hydrogels in CaCl_2_ solution were also fitted typical pseudo-second order kinetics model. Figure 10 described the plots for pseudo-first-order and pseudo-second-order kinetics of γ-PGA/ε-PL hydrogels in N_1_, N_2_, and N_3_ (N_1_: 0.0154 mol/L, N_2_: 0.0308 mol/L, and N_3_: 0.154 mol/L). Table 3 lists the kinetic parameters of the pseudo-first-order and pseudo-second-order models. Compared with the pseudo-first-order, it shows that the pseudo-second-order model presents a relatively higher correlation coefficient (*R*_adj_^2^ > 0.99), and it has a closer experimental swelling degree (*Q*_e,e_) and calculated swelling degree (*Q*_e,c_). This indicates that the adsorption kinetic of γ-PGA/ε-PL hydrogels in CaCl_2_ solution follow the pseudo-second-order model and shows that the rate-limiting step of swelling in CaCl_2_ solution was chemisorption and chelation involving valence forces through the sharing or exchange of electrons between the γ-PGA/ε-PL hydrogels and ions [42].

##### 3.2.2.4. The Effect of CrCl_3_ Concentration

**Figure 11:**
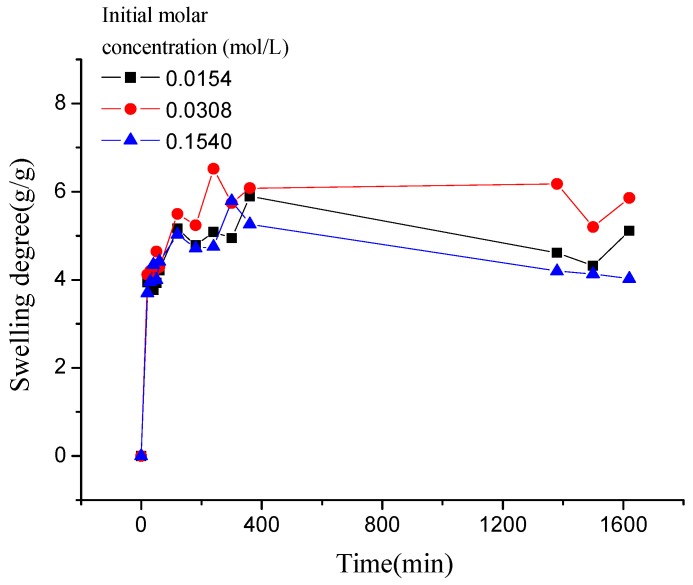
The swelling kinetics of γ-PGA/ε-PL hydrogels in CrCl_3_ solution.

As shown in Figure 11, compared to the swelling kinetics of γ-PGA/ε-PL hydrogels in the above two kinds of ions solution, the swelling kinetics of hydrogels in CrCl_3_ solution showed different trends. In less than 120 min, the swelling degree increased rapidly, compared to that observed in NaCl and CaCl_2_ solutions. However, when γ-PGA/ε-PL hydrogels swelled from 120 min to 360 min, the swelling degrees appeared to irregularly rise and fall. After more than 360 min, the swelling degrees were decreased to about 1.15 g/g because the Cr^3+^ has a stronger charge shielding effect than Na^+^ and Ca^2+^. When the γ-PGA/ε-PL hydrogel-adsorbing Cr^3+^ solution reached the critical point, the stronger charge attraction force between Cr^3+^ and –COO^−^ maintained a balance with the total of repulsive force between –COO^−^ and other forces. Then, the charge attraction forces between Cr^3+^ and –COO^−^ became stronger because the hydrogels adsorbed more ions, which collapsed the network structure of the hydrogels [43].

### 3.3. The Desorption Kinetic

**Figure 12:**
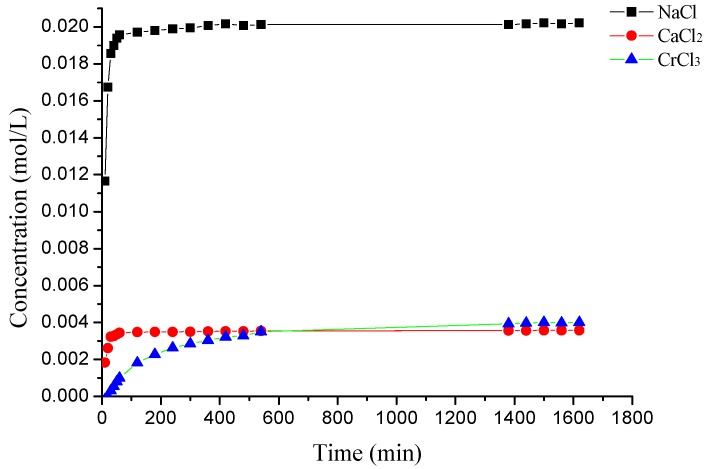
The desorption kinetics of γ-PGA/ε-PL hydrogels in ion solutions.

**Figure 13:**
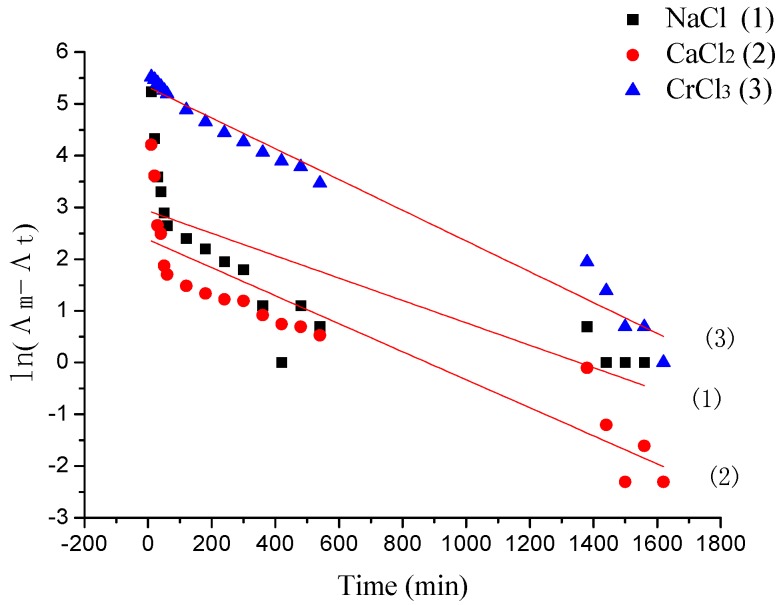
Pseudo-first-order kinetic plot for the desorption of γ-PGA/ε-PL hydrogels in NaCl, CaCl_2_, and CrCl_3_ solutions.

The desorption rate of γ-PGA/ε-PL hydrogels in NaCl, CaCl_2_, and CrCl_3_ solutions at different times is shown in Figure 12. In previous studies, the desorption process of some ions from hydrogels obeyed pseudo-first-order kinetics [35]. However, as shown in Figure 13 and Table 4, the pseudo-first-order model presented relatively lower correlation coefficients (*R*_adj_^2^ < 0.90) in NaCl and CaCl_2_ solutions, and they had different values between experimental conductivity (Λ_m,m_) and calculated conductivity (Λ_m,c_). This might due to the appearance of re-adsorption ions of hydrogels in the desorption media. Before 60 min, desorption capacities of ions increased rapidly. However, the release rate of Na^+^ was the highest in the whole solutions (the concentration increased from 0.01157 mol/L to 0.01948 mol/L). The uptake rate decreased after that. This might also be due to the charge shielding effect of ions in the hydrogel’s network. In deionized water, when the hydrogels containing adsorbed ions were subjected to water molecules, the ions on the surface of hydrogels readily hydrated and desorbed from the hydrogel as they weakly interacted on the surface, which was attributed to the initial rapid increase in conductivity that, later on, slows down as time proceeds. The hydration reduced the stiffness of cross-linked units with the hydrogel and that weakened the interactions between the adsorbed ions and the cross-linked units, which led to desorption of ions from the hydrogel’s network.

## 4. Conclusion

In this study, novel γ-PGA/ε-PL hydrogels were successful prepared by the EDC/NHS mediated system. The pH significantly influenced the swelling capacities of γ-PGA hydrogels. The swelling capacity of γ-PGA/ε-PL hydrogels decreased with the increase of the ion concentration. The swelling kinetics presented that γ-PGA/ε-PL hydrogels were ion-sensitive and showed a more limited swelling degree in ion solutions with higher ionic valence numbers. The swelling degrees of γ-PGA/ε-PL hydrogels in NaCl, CaCl_2_, and CrCl_3_ solutions were 54.91, 9.94, and 4.86 g/g, respectively. Additionally, the swelling kinetics of γ-PGA/ε-PL hydrogels showed that the pseudo–second order kinetic model presented relatively higher linear correlation coefficients (over 0.99) in NaCl and CaCl_2_ solutions. The desorption capacities of ions increased rapidly with time (before 60 min) and the release rate of Na^+^ was at its maximum in various solutions (0.01948 mol/L). 

On the whole, γ-PGA/ε-PL hydrogels presented ionic sensitivity and desorption of ions. Swelling and desorption kinetics indicated that γ-PGA/ε-PL hydrogels might be used in some key applications, such as drug release, absorbents, ion-sensitive smart materials, etc. 

## Figures and Tables

**Table 1:** The morphology of γ-PGA/ε-PL hydrogels after swelling equilibrium in 0.1540 mol/L NaCl, CaCl_2_, and CrCl_3_ solutions.

	NaCl	CaCl_2_	CrCl_3_
Form	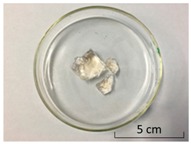	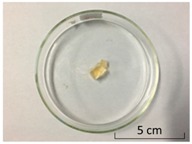	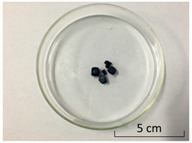
Cross-section	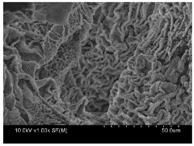	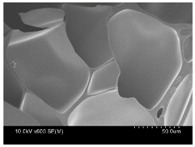	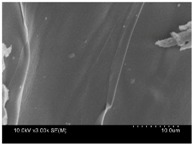
Surface	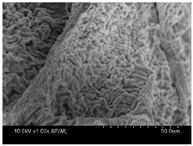	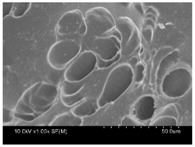	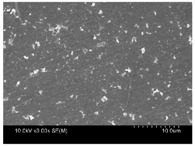

**Table 2:** Constants and correlation coefficients of the two kinetic models for Na(I) adsorption onto γ-PGA/ε-PL hydrogels (N_1_: 0.0154 mol/L, N_2_: 0.0308 mol/L, N_3_: 0.154 mol/L).

	*Q*_e,e_ (g/g)	Pseudo-First-Order			Pseudo-Second-Order		
		*K*_1_ (min^−1^)	*Q*_e,c_ (g/g)	*R*_adj_^2^	*K*_2_ (min^−1^)	*Q*_e,c_ (g/g)	*R*_adj_^2^
N_1_	59.12	0.00293	48.68	0.97646	0.000110385	63.61	0.9983
N_2_	50.80	0.00105	41.01	0.97574	0.000105524	47.24	0.99106
N_3_	26.42	0.00104	18.66	0.93623	0.000333858	24.49	0.9931

**Table 3:** Constants and correlation coefficients of the two kinetic models for Ca(II) adsorption onto γ-PGA/ε-PL hydrogels (N1: 0.0154 mol/L, N2: 0.0308 mol/L, N3: 0.154 mol/L).

	*Q*_e,e_ (g/g)	Pseudo-First-Order			Pseudo-Second-Order		
		*K*_1_ (min^−1^)	*Q*_e,c_ (g/g)	*R*_adj_^2^	*K*_2_ (min^−1^)	*Q*_e,c_ (g/g)	*R*_adj_^2^
N_1_	9.94	0.00344	2.13	0.63707	0.005619146	10.14	0.99965
N_2_	8.15	0.00091	1.99	0.34821	0.012211097	7.49	0.99345
N_3_	6.33	0.0018	1.47	0.79817	0.013841741	6.30	0.99973

**Table 4:** Constants and correlation coefficients of pseudo-first-order kinetic models for NaCl (1), CaCl_2_ (2), and CrCl_3_ (3) adsorption onto γ-PGA/ε-PL hydrogels.

	Λ_m,m_ (μs)	Pseudo-First-Order		
		*K*_1_ (min^−1^)	Λ_m,c_ (μs)	*R*_adj_^2^
(1)	2358.5	0.00216	2185.29	0.57959
(2)	832.3	0.00271	822.59	0.82502
(3)	1409.56	0.00297	1400.79	0.97701
